# Supplementary material for: L-shaped association between the blood pressure response index and short-term mortality in intensive care patients with sepsis: An analysis based on the MIMIC-IV database
Source: PLoS One. 2025 Jun 11;20(6):e0325110. doi: 10.1371/journal.pone.0325110 (PMC12157094; doi:10.1371/journal.pone.0325110)
Supplement: Attachment — Submitted filename: Response to Reviewers.docx [file pone.0325110.s002.docx]

Dear Dr. Aljamaan,

Thank you very much for your email and for the constructive feedback on our manuscript entitled **"L-Shaped Association between the Blood Pressure Response Index and Short-Term Mortality in Intensive Care Patients with Sepsis: An Analysis Based on the MIMIC-IV Database." (Manuscript PONE-D-24-45268)** We appreciate the time and effort the reviewers and you have put into evaluating our work.

We are grateful for the opportunity to revise our manuscript and address the points raised during the review process. We will carefully review and incorporate the suggestions to improve the manuscript, ensuring that we meet PLOS ONE publication criteria.

We will include the following items with the resubmission:

A rebuttal letter responding to each point raised by the academic editor and reviewer(s), labeled ‘Response to Reviewers.’

A marked-up version of the manuscript highlighting the changes made, labeled ‘Revised Manuscript with Track Changes.’

An unmarked version of the manuscript without tracked changes, labeled ‘Manuscript.’

Should we need to update any financial disclosures or other necessary information, we will include the revisions in our cover letter.

Once again, we appreciate the opportunity to improve our manuscript. We look forward to submitting the revised version and hope to meet your expectations.

Kind regards,

Heping Xu, PhD

Department of Emergency Medicine

Hainan General Hospital / Hainan Affiliated Hospital of Hainan Medical University

Haikou City, Hainan Province, 570311

Email: xhp21528@163.com

November 12, 2024

**Response to Editor:
Journal Requirements:**

1. Please ensure that your manuscript meets PLOS ONE's style requirements, including those for file naming.

**Response:**

The manuscript has been formatted to comply with PLOS ONE style requirements. The documents have been renamed and formatted as per the provided guidelines.

2. We note that you have indicated that there are restrictions to data sharing for this study. For studies involving human research participant data or other sensitive data, we encourage authors to share de-identified or anonymized data. However, when data cannot be publicly shared for ethical reasons, we allow authors to make their data sets available upon request. For information on unacceptable data access restrictions, please see http://journals.plos.org/plosone/s/data-availability#loc-unacceptable-data-access-restrictions.

Before we proceed with your manuscript, please address the following prompts:

a) If there are ethical or legal restrictions on sharing a de-identified data set, please explain them in detail (e.g., data contain potentially identifying or sensitive patient information, data are owned by a third-party organization, etc.) and who has imposed them (e.g., a Research Ethics Committee or Institutional Review Board, etc.). Please also provide contact information for a data access committee, ethics committee, or other institutional body to which data requests may be sent.

**Response:**

Thank you for your valuable comments regarding the data availability for our manuscript. We appreciate your attention to this important matter.

The data used in our study is derived from the MIMIC-IV database, which is an anonymized public database approved by the institutional review boards of the Massachusetts Institute of Technology (MIT) and Beth Israel Deaconess Medical Center (BIDMC). The requirement for informed consent was waived due to the thorough anonymization and de-identification of all patient information in the database. However, due to the sensitive nature of the data, direct sharing of the dataset is not permissible.

The datasets generated and/or analyzed during the current study are available through the MIMIC-IV database (https://mimic.physionet.org/). Access is granted via the PhysioNet platform (https://physionet.org/content/mimiciv), subject to registration and completion of the required CITI program training in human subjects research. Interested researchers can obtain the data directly from PhysioNet following the necessary approval process.

We hope this clarifies the situation regarding data access. Please feel free to contact us if you need further details or have any additional questions.

Thank you once again for considering our manuscript for publication in PLOS ONE.

**revised content: Please see red fonts on Page 18，line 452-459.**

“The datasets analyzed in this study are available in the MIMIC-IV database (https://mimic.physionet.org/), an anonymized resource approved by the IRBs of the Massachusetts Institute of Technology (MIT) and Beth Israel Deaconess Medical Center (BIDMC), with informed consent waived. Due to the sensitive nature of the data, direct sharing is not permitted. Researchers can access and request the data through the PhysioNet platform (https://physionet.org/content/mimiciv) after completing registration and CITI training.”

3.Your ethics statement should only appear in the Methods section of your manuscript. If your ethics statement is written in any section besides the Methods, please move it to the Methods section and delete it from any other section. Please ensure that your ethics statement is included in your manuscript, as the ethics statement entered into the online submission form will not be published alongside your manuscript.

**Response: The ethics statement has been moved to the Methods section and removed from any other sections. Please see red fonts on Page 6，line 147-151.**

**Additional Editor Comments:**

Before proceeding with your paper for decision about revision, you need to enrich the introduction part and explain the rationale and the literature and evidence support physiological and by literature for the newly proposed definition used for the BPRI (MAP divided by VIS)

**Response:** Thank you very much for your valuable feedback. In response to your suggestion, we have reorganized and expanded the introduction to enhance its depth and clarity. Specifically, we have provided a more detailed explanation of the physiological rationale and literature supporting the newly proposed definition for BPRI (MAP ÷ VIS). These revisions are highlighted in red in the introduction section, between **pages 3 and 4, lines 63–105.**

**revised content:**

Septic shock is a critical and often fatal manifestation of sepsis, characterized by life-threatening organ dysfunction resulting from a dysregulated host response to infection (1). It is one of the leading causes of mortality in intensive care unit (ICU) patients, with over a quarter of septic shock patients dying despite intensive treatment (2, 3). One of the key components of septic shock management is the use of vasoactive drugs, which aim to restore hemodynamic stability. However, the physiological mechanisms underlying the cardiovascular responses to vasoactive therapy in septic patients remain incompletely understood, limiting the ability to predict clinical outcomes and guide therapeutic decisions (1).

Sepsis is known to induce a complex cascade of changes in vascular tone, leading to hypotension and impaired tissue perfusion. Although cardiac output may remain stable in the early stages of septic shock, dysregulation of arterial tone leads to uneven blood flow distribution, resulting in suboptimal organ perfusion and contributing to multi-organ dysfunction (4, 5). Evaluating arterial tone is crucial for understanding these mechanisms, and several approaches have been proposed. For instance, the Windkessel model uses the ratio of left ventricular end-systolic pressure (LVESP) to stroke volume (SV) as an index of effective arterial elastance (6), while pulse contour analysis provides a method for assessing LVESP (7). These techniques, however, are invasive and not well-suited for routine clinical use, particularly in settings where rapid, real-time assessments of vascular reactivity are needed.

Recent studies have sought to identify more accessible and non-invasive methods for assessing vascular function in septic shock. One promising approach is the Blood Pressure Reactivity Index (BPRI), introduced by Yujie Chen et al. (8), which has been proposed as a potential predictor of prognosis in septic shock patients. However, the original definition of BPRI uses the vasoactive-inotropic score (VIS), which does not account for all vasoactive drugs commonly used in clinical practice, such as phenylephrine. This limitation restricts the BPRI's clinical utility and its ability to fully reflect the physiological responses to vasoactive therapy. Additionally, the relationship between BPRI and short-term mortality, a key outcome in septic shock, has not been thoroughly investigated.

To address these gaps, we propose a refined definition of BPRI by dividing the mean arterial pressure (MAP) at a specific time point by the vasoactive-inotropic score (VIS). This new index is designed to offer a more comprehensive and clinically relevant measure of vascular reactivity by considering the combined effects of all vasoactive drugs, including those not previously incorporated into the VIS. By providing real-time, easily accessible data on vascular status, this revised BPRI could serve as a valuable tool for assessing the effectiveness of vasoactive therapy and predicting outcomes in septic shock patients.

The aim of this study is to investigate the relationship between the newly proposed BPRI at ICU admission and key clinical outcomes, including in-hospital mortality, 28-day mortality, and 90-day mortality, in patients with septic shock. We hypothesize that the revised BPRI will be a reliable predictor of short-term mortality, helping to guide clinical decision-making and improve patient prognostication in septic shock.
